# Sustained Delivery Growth Factors with Polyethyleneimine‐Modified Nanoparticles Promote Embryonic Stem Cells Differentiation and Liver Regeneration

**DOI:** 10.1002/advs.201500393

**Published:** 2016-04-08

**Authors:** Meiyan Wang, Xiaomei Yang, Peng Zhang, Lei Cai, Xibin Yang, Youwei Chen, Yuanya Jing, Jilie Kong, Xiaowei Yang, Fang‐Lin Sun

**Affiliations:** ^1^ Research Center for Translational Medicine at East HospitalSchool of Life Sciences and TechnologyTongji UniversityShanghai200120/200092P.R. China; ^2^ Department of PharmaceuticsSchool of PharmacyFudan UniversityShanghai201203P. R. China; ^3^ Bio‐X InstitutesKey Laboratory for the Genetics of Developmental and Neuropsychiatric Disorders (Ministry of Education)Shanghai Key Laboratory of Psychotic Disorders (No. 13dz2260500)Shanghai Jiaotong UniversityShanghai200240P.R. China; ^4^ Department of Chemistry and Institutes of Biomedical SciencesFudan UniversityShanghai200433P.R. China; ^5^ School of Materials Science and EngineeringTongji UniversityShanghai200092P.R. China

**Keywords:** embryonic stem cells, growth factor delivery, hepatocyte‐like cell, polyethyleneimine (PEI), silica nanoparticle

## Abstract

Stem‐cell‐derived hepatocyte transplantation is considered as a potential method for the therapy of acute and chronic liver failure. However, the low efficiency of differentiation into mature and functional hepatocytes remains a major challenge for clinical applications. By using polyethyleneimine‐modified silica nanoparticles, this study develops a system for sustained delivery of growth factors, leading to induce hepatocyte‐like cells (iHeps) from mouse embryonic stem cells (mESCs) and improve the expression of endoderm and hepatocyte‐specific genes and proteins significantly, thus producing a higher population of functional hepatocytes in vitro. When transplanted into liver‐injured mice after four weeks, mESC‐derived definitive endoderm cells treated with this delivery system show higher integration efficiency into the host liver, differentiated into iHeps in vivo and significantly restored the injured liver. Therefore, these findings reveal the multiple advantages of functionalized nanoparticles to serve as efficient delivery platforms to promote stem cell differentiation in the regenerative medicine.

## 1 Introduction

This is an open access article under the terms of the Creative Commons Attribution License, which permits use, distribution and reproduction in any medium, provided the original work is properly cited.

Hepatic failure including cirrhosis and fibrosis is a serious threat to human health, and recently embryonic stem cells (ESCs) have been recognized as promising therapeutic agents for the treatment of acute liver failure and advanced cirrhosis.^1^, ^2^ Both murine and human ESCs were reported to differentiate into hepatocytes or hepatocyte‐like cells successfully.^3^, ^4^, ^5^, ^6^, ^7^ As known, most of the current protocols used embryoid bodies (EBs) and simple adherent monolayer cultures with soluble growth factors and small chemicals to differentiate ESCs into hepatocytes; however, they are still subjected to inefficient differentiation and low purity of functional hepatocytes.^8^, ^9^ The differentiation of stem cell could usually be affected by not only the internal factors, but also the exogenous growth factors, however, these substances tend to have short half‐lives and are needed to externally add to the culture media in order to maintain the effective level during the whole process.^10^ While higher concentrations of added growth factors can induce cell toxicity.^11^ Therefore, developing a stable and highly effective differentiation system with sustained delivery of growth factors is of the utmost importance.

Recently, the application of biomaterials in the growth factors delivery system of stem cell has been reported.^12^ The polymer‐based delivery systems have been developed to date, including poly(lactide‐*co*‐glycolide) (PLGA), polylactic acid (PLA), and polycaprolactone. PLGA particles were used as a delivery system to package growth factors within EBs to improve the differentiation of human embryonic stem cells into the vascular or osteogenic lineage.^13^, ^14^ However, remaining organic solvents used in the fabrication of polymer nanoparticles may adversely affect sensitive protein bioactivity and the loading efficiency of larger biomolecules, such as growth factors.^15^, ^16^

During the past decade, inorganic nanomaterial‐based carriers were widely used to deliver genes, drugs, and bioactive molecules due to their ability to overcome biological barriers and deliver larger biomolecules, leading to eliminate the loss of biological activity and likely enhance long‐term sustained release of bioactive molecules. Among various inorganic nanomaterials, silica nanoparticles have attracted significant attention as a superior multifunctional nanomaterial because of its biocompatibility, tunable pore volumes, unique interfacial features, and easily functionalized surfaces.^17^, ^18^ In order to maximize loading capacity and improve the suspension stability or processability of delivery system, the surface of silica materials should be modified. One of the most attractive groups available for surface modification is the amino group by using amino silanes or cationic polymers such as polyethyleneimine (PEI). Positively charged PEI‐coated silica particles could dramatically enhance their dispersion stability and prevent the aggregation at physiological pH compared to corresponding bare materials, thus nowadays silica modified with PEI emerged as promising alternative delivery systems of biological molecules, such as nucleic acids, enzymes, and antibodies.^19^, ^20^, ^21^ Chen et al. demonstrated that positively charged mesoporous silica nanoparticle (MSNs) delivering hepatocyte nuclear factor 3β plasmid DNA could promote the production of hepatocyte‐like cells from induced pluripotent stem cells.^22^ MSNs modified with PEI were also reported to facilitate DNA and siRNA delivery because of the large number of amine groups on PEI, enhance particle uptake into cells through rapid endocytosis, and promote endosomal escape.^19^,^23^, ^24^, ^25^ Compared to unmodified silica particles, PEI as the polymer coating to the surface of MSNs not only enhances suspension stability of MSNs but also promotes bonding growth factors through electrostatic interactions and hydrogen bonding between amino groups of the MSNs and functional groups of the growth factors,^26^, ^27^ which may improve growth factors loading, stabilize growth factors, and extend the time of cells exposure to the growth factors.

**Scheme 1:**
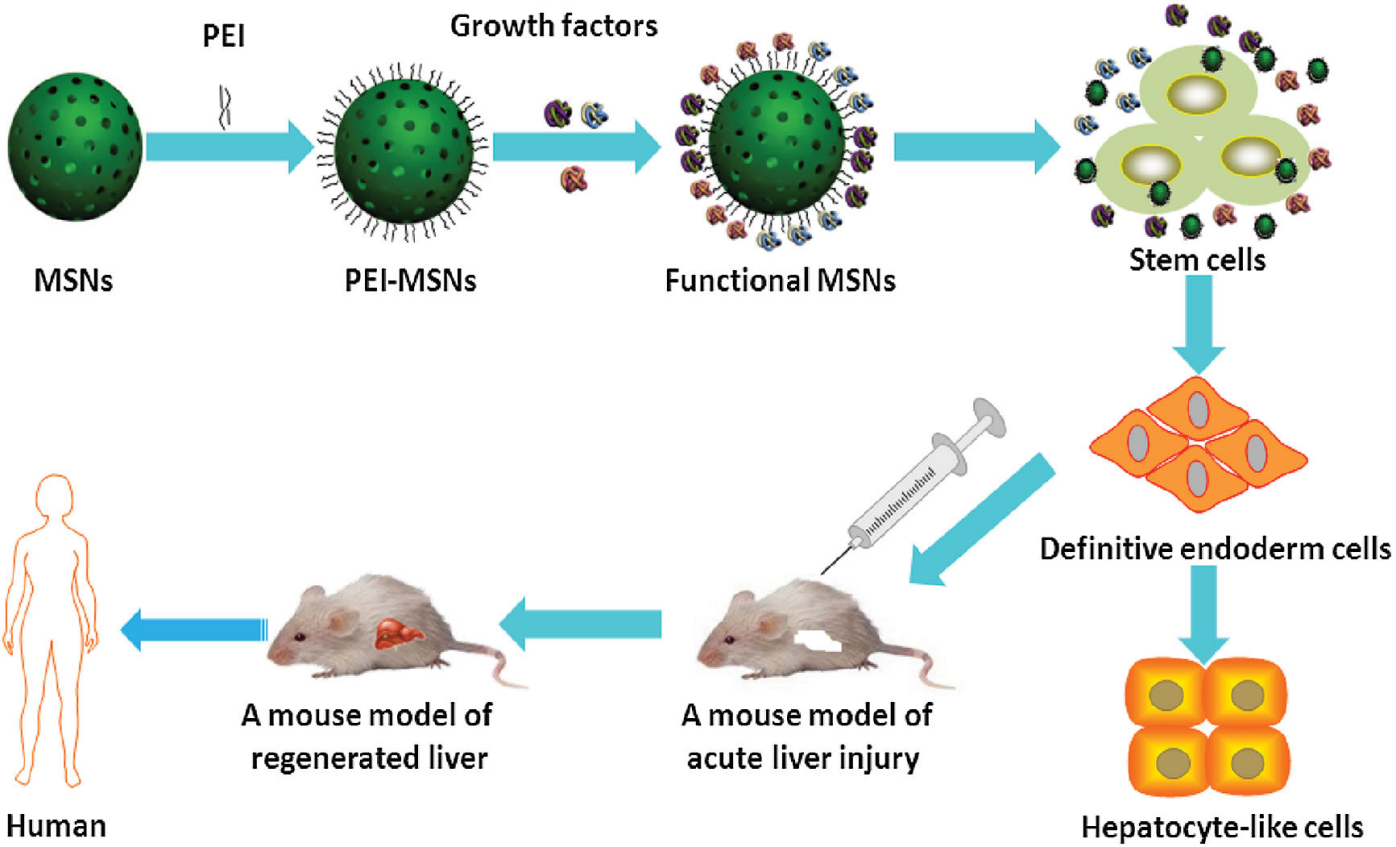
Schematic representation of polyethyleneimine (PEI)‐modified mesoporous silica nanoparticles (MSNs) for directed differentiation of mouse embryonic stem cells (mESCs) into hepatocyte‐like cells (iHeps) in vitro and in vivo. Growth factors were adsorbed on the PEI‐MSNs to form a GF‐PEI‐MSN complex. The mESCs induced by GF‐PEI‐MSN complexes exhibited significantly improved differentiation toward hepatocyte‐like cells with mature functions in vitro, and the induced cells reconstitute damaged hepatic tissues after transplantation in vivo.

In our study, we established an inorganic/polymer culture system for sustained promoting the differentiation of mouse embryonic stem cells (mESCs) into hepatocyte‐like cells by using silica nanoparticles modified with hyperbranched PEI as the carrier for exogenous growth factors (**Scheme** **1**). We found that PEI‐modified MSNs (PEI‐MSNs) could be internalized by the mESCs sustained delivery growth factors without impairing cell viability and efficiently deliver growth factors, such as Activin A, acidic fibroblast growth factor (aFGF) and hepatocyte growth factor (HGF), which were reported to play an important role in hepatic differentiation from the ESCs.^5^, ^28^, ^29^ Furthermore, after three‐day treatment with growth factor (GF)‐PEI‐MSNs, predifferentiated definitive endoderm (DE) cells were sufficient to induce more robust differentiation of hepatocyte‐like cells in vivo upon transplantation into a mouse model of chronic liver injury. Moreover, the injured livers were more efficiently restored compared with the controls, which attributed to transplanted cells and the paracrine effect of predifferentiated cells. Thus, this treatment may offer a potential approach for regenerative medicine applications, especially in the treatment of liver diseases.

## 2 Results and Discussion

### 2.1 Characterization of MSNs and PEI‐Modified MSNs

**Figure 1:**
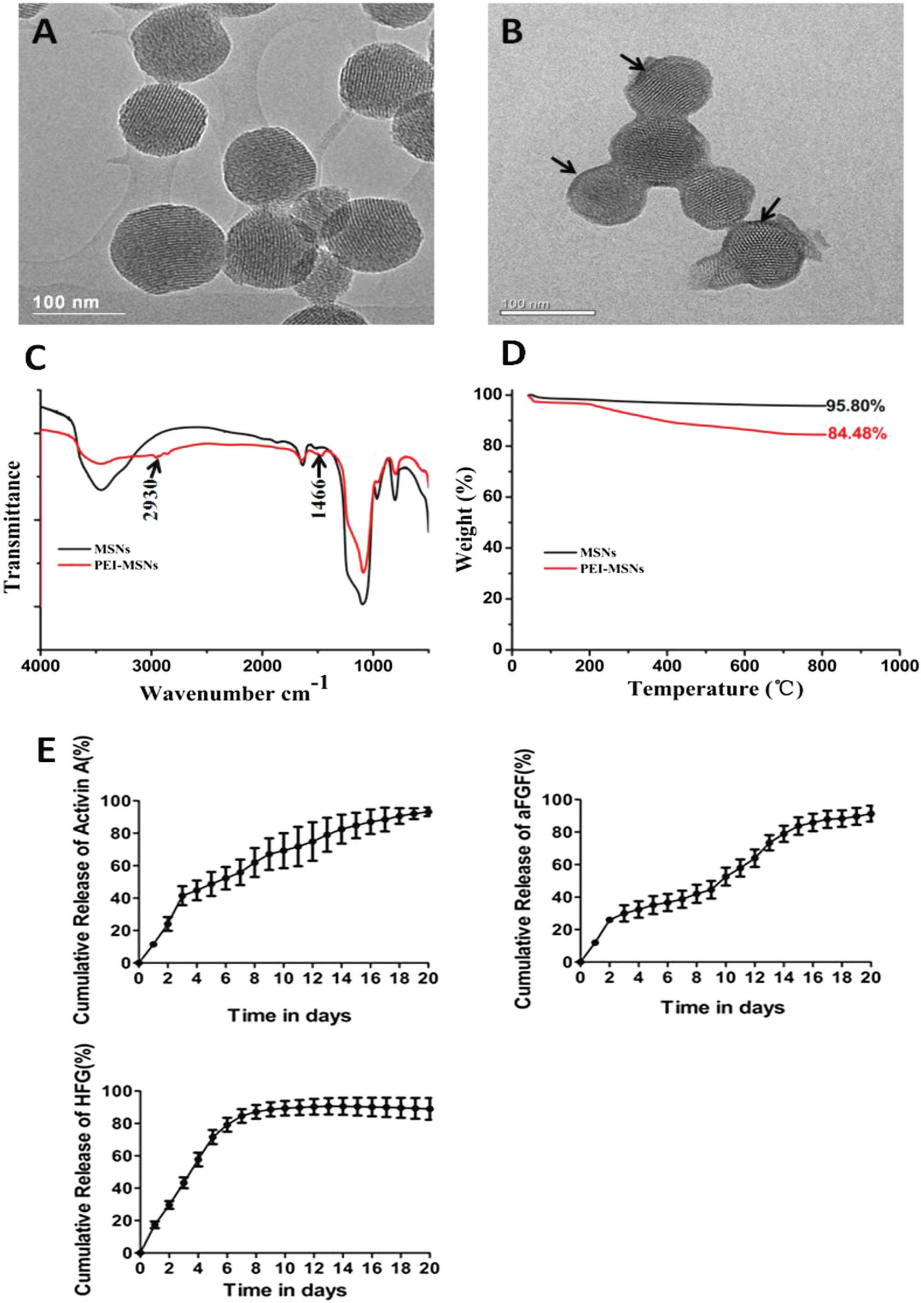
TEM images of A,B) mesoporous silica nanoparticles (MSNs) and PEI‐modified mesoporous silica nanoparticles (PEI‐MSNs), black arrow: PEI coating; C) FT‐IR spectrum; D) Thermogravimetric analysis (TGA) curves of the MSNs and PEI‐MSNs, and E) The cumulative release profiles of PEI‐MSNs loaded with Activin A, aFGF, and HGF. Data are presented as means ± SD (spontaneous differentiation (control)).

Silica nanoparticles were synthesized by using a previously described method,^30^, ^31^ followed by modification with PEI to obtain amino‐functionalized MSNs. Transmission electron microscope (TEM) images of these MSNs (**Figure** **1**A) exhibited well‐ordered hexagonal mesopores even after PEI modification. In addition, PEI‐modified MSNs appear a thin polymer film covering the whole surface of MSNs ((Figure 1B). The average hydrodynamic size of these MSNs was ≈110 nm as measured by dynamic light scattering and increased to about 210 nm for the PEI‐MSNs in phosphate‐buffered saline (PBS) (Table S1, Supporting Information). With the large number of amine groups for PEI, the surface *ζ* potential of PEI‐coated MSNs changed from strongly negative to positive (Table S1, Supporting Information), which provides PEI‐MSNs with a platform for the delivery of growth factors.

The Fourier transform infrared (FT‐IR) spectrum of the PEI‐MSNs displayed absorption band at 1466 and 2930 cm^−1^, attributed to the stretching vibration of C=N and C—H, indicating the presence of amine surface functional groups on the surface of the particles, which could interact with the growth factors. In contrast, the MSNs did not show obvious absorption peak in these wave numbers (Figure 1C). In addition, the amount of PEI in the MSNs was estimated by thermogravimetric analyses (TGA). MSNs and PEI‐MSNs showed a weight loss of 4.2 and 15.5 wt%, respectively (Figure 1D), showing the adsorbed PEI content at ≈11.3 wt%. As shown in Table S2 (Supporting Information), after functionalization with PEI, specific surface areas (*a*_s_, Brunauer–Emmett–Teller(BET)) of MSNs significantly decreases, while the pore volumes and pore size for PEI‐MSNs is similar to that of MSNs, meaning that PEI molecules were successfully adsorbed onto the MSNs. Furthermore, obvious cytotoxicity of MSNs or PEI‐MSNs was not observed using a CCK‐8 assay, indicating that the synthesized nanoparticles could serve as excellent carriers (Figure S1, Supporting Information).

### 2.2 Loading and Release Evaluation of Growth Factor on PEI‐MSNs

To further improve the differentiation efficiency of every step of hepatic differentiation (mESCs to DE cells, DE cells to hepatoblasts and hepatoblasts to hepatocyte‐like cells), we investigated the delivery of key growth factors that regulate hepatogenesis, including Activin A, aFGF, and HGF. Activin A has been reported to induce definitive endoderm differentiation of ESCs;^10^, ^32^ DE cells constitute the embryonic germ layer that produces hepatic cells;^33^ aFGF and HGF are essential for liver development, with aFGF efficiently initiating hepatic differentiation of ESCs from the definitive endoderm and HGF promoting hepatic growth.^34^, ^35^ The novel growth factors delivery system was prepared by mixing Activin A, aFGF, and HGF with positively charged PEI‐MSNs. After the growth factors were added, the hydrodynamic diameters of the growth‐factor‐loaded PEI‐MSN composites increased to larger sizes than before, while the *ζ*‐potential of these complexes decreased slightly. This indicates that the growth factors were successfully attached to the positively charged PEI‐MSNs, possibly through electrostatic interactions and hydrogen bonding between amino groups of PEI and functional groups of the growth factors, which allowed the growth factors to be released more readily in the biological system as reported previously.^36^ In addition, the loading efficiency of Activin A, aFGF and HGF on the PEI‐MSNs was ≈82.23% ± 7.98%, 81.30% ± 0.18%, and 77.91% ± 4.57%, respectively. The in vitro release profiles of Activin A, aFGF, and HGF from the PEI‐MSN in PBS are shown in Figure 1E. Activin A release started with an initial burst followed by a slow release without an evident plateau, which is indicative of ongoing release thereafter. aFGF release pattern started with an initial burst followed by a decline phase which entered a steady‐state release. HGF release started with an initial burst and then was followed by a steady‐state release. Therefore our results show that PEI‐MSNs were considered as a novel system for long‐term delivery of the growth factors.

### 2.3 PEI‐MSNs Loaded with Growth Factors Facilitate mESCs Differentiation into Hepatocyte‐Like Cells

**Figure 2:**
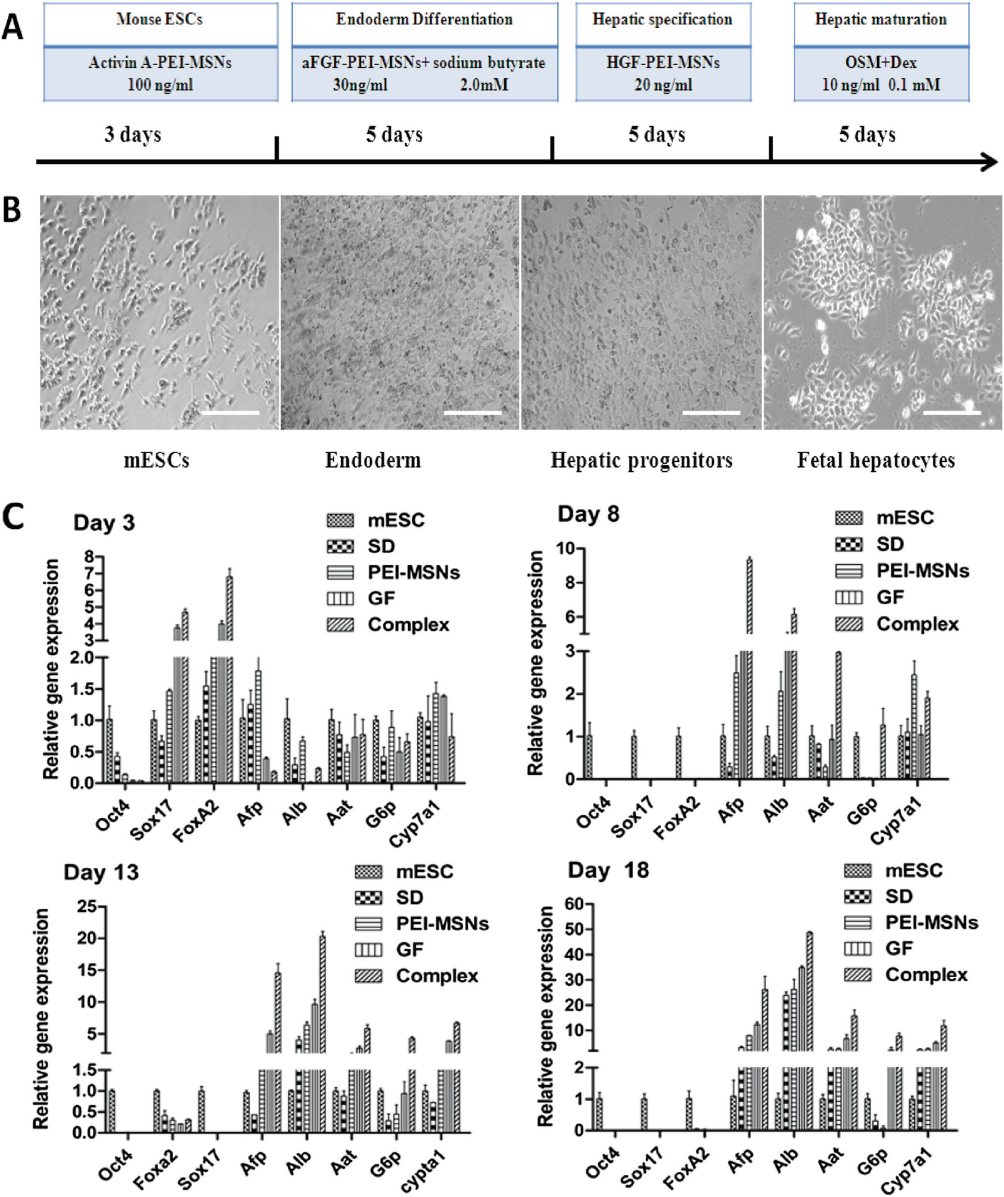
A) Schematic representation of the developed multistep differentiation procedure. B) Phase contrast images of differentiating cells at days 3, 8, 13, and 18. Scale bar: 200 μm C) Temporal expression patterns of genes by qPCR analysis during the induction of mESCs treated with GF‐PEI‐MSN complexes, PEI‐MSNs and growth factors alone or treatment without PEI‐MSNs and any growth factors. Data were calculated in relation to the expression of the housekeeping gene GAPDH (glyceraldehyde phosphate dehydrogenase) and used as an internal standard and are shown as the expression relative to that of the undifferentiated mESCs using the comparative CT Method (2^−CT^). Results represent the mean ± SD (*n* = 3).

We investigated whether PEI‐MSNs loaded with growth factors could promote the differentiation of mESCs into hepatocyte‐like cells according to the protocol described in **Figure** **2**A,B. A quantitative polymerase chain reaction (qPCR (polymerase chain reaction)) analysis was performed at various times during the culture process to determine the degree to which the mESCs differentiated toward a hepatocyte‐like cell phenotype. As shown in Figure 2C, the expression of pluripotent marker octamer‐binding transcription factor 4 (Oct4)^37^ gradually decreased with time, and a significantly greater reduction in mESCs was induced by growth‐factor‐loaded PEI‐MSN complexes compared with other groups (mESCs induced by PEI‐MSNs, growth factors alone, or spontaneously differentiated mESCs), which indicated a more rapid loss of stemness during differentiation of the mESCs treated with GF‐PEI‐MSN complexes. The expression of DE transcription factor markers SRY‐box containing gene 17 (Sox1)^38^ and forkhead box A2 (FoxA2)^39^ increased within 3 d of the differentiation process and slowly diminished thereafter in all groups; however, the levels were significantly higher in the mESCs treated with GF‐PEI‐MSN complexes compared with other groups. The gene expression of hepatocyte‐related markers, such as alpha‐fetoprotein (AFP)^40^ and albumin (ALB),^40^, ^41^ was strongly upregulated in the mESCs containing GF‐PEI‐MSN complexes from day 8 compared with the other three groups. The mRNA expression of hepatic functional markers and hepatic metabolic enzyme genes, such as α‐1‐antitrypsin (AAT),^42^ glucose‐6‐phosphatase (G6P)^43^ and members of the cytochrome P450 subunit CYP7A1,^44^ showed a time‐dependent upregulation from day 13 and were highly expressed at day 18 in the hepatocyte‐like cells that were induced by the mESCs treated with GF‐PEI‐MSN complexes.

**Figure 3:**
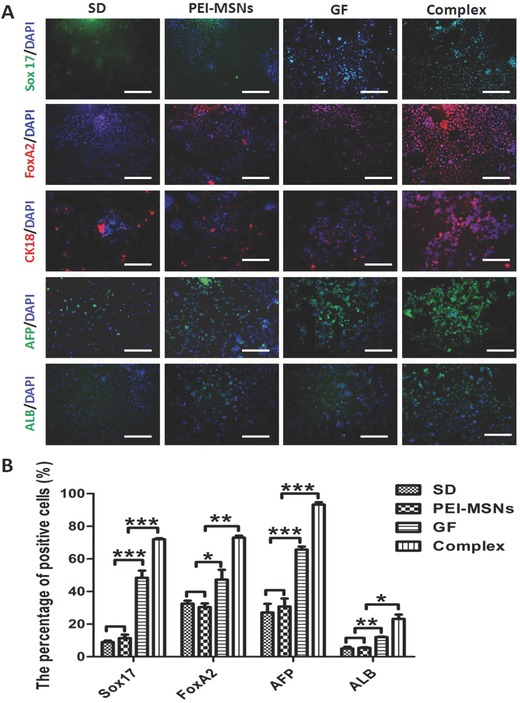
Immunofluorescence and flow cytometry analysis of the cellular stage‐specific protein expression in differentiating cells in vitro at days 3 and 18 induced by GF‐PEI‐MSN complexes, PEI‐MSNs, growth factors alone, and without treatment. A) Fluorescent images of definitive endoderm (DE) cell markers Sox17 (green) and FoxA2 (red) and liver‐specific markers CK18 (red), AFP (green), and ALB (green) acquired using an inverted fluorescence microscope. Nuclei were counterstained with DAPI (blue). Scale bar: 200 μm. B) Quantitative depiction of flow cytometric analysis for Sox17, FoxA2, AFP, and ALB expression in the four groups. All data are represented as the mean ± SD (*n* = 3). *P < 0.05, **P < 0.01, and ***P < 0.001.

To confirm the in vitro hepatic differentiation of the mESCs and expression of the endoderm‐specific markers Sox17 and FoxA2 at day 3 and the liver‐specific markers cytokeratin 18 (CK18), AFP, and ALB at day 18, immunofluorescence staining was used to detect these markers (**Figure** **3**A and single channel images shown in Figure S2 in the Supporting Information). There was a greater upregulation of the expression of these markers by GF‐PEI‐MSN complexes in the induced cells compared with the other three groups, consistent with the results of the qPCR analyses.

To further quantify the expression levels associated with DE and hepatocyte‐like cells, the mESC‐derived cells were analyzed for the expression of Sox17 and FoxA2 at day 3 and expression of AFP and ALB at day 18 using flow cytometry analysis (Figure 3B; Figure S3, Supporting Information). The percentages of Sox17, FoxA2, AFP and ALB positive cells in the spontaneously differentiated cells and directly differentiated cells by PEI‐MSNs, growth factors alone, and GF‐PEI‐MSN complexes were shown in **Table** **1**. These results clearly demonstrated that the percentages of cells positive for Sox17, FoxA2, AFP, and ALB in the directly differentiated cells treated with GF‐PEI‐MSN complexes were significantly higher than in the other groups (Figure 3B). These data demonstrate that more hepatic differentiation resulted from the treatment with GF‐PEI‐MSN complexes and the delivery of growth factors by PEI‐MSNs can provide an efficient platform for improving definitive hepatic differentiation in vitro. This may be attributed to increasing the growth factor concentration and bioavailability or extending time course for sustainable release in GF‐PEI‐MSN complexes group.

**Table 1:** The percentages of positive cells in the differentiated cells of different treatment

Positive cells name	Control (without treatment) [%]	PEI‐MSNs [%]	Growth factors [%]	GF‐PEI‐MSN complexes [%]
Sox17	8.97 ± 0.90	11.35 ± 2.17	48.40 ± 4.36	72.03 ± 0.60
FoxA2	32.53 ± 1.88	30.47 ± 2.27	47.33 ± 5.98	73.00 ± 1.23
AFP	27.10 ± 5.35	30.80 ± 4.94	65.73 ± 1.79	93.27 ± 1.42
ALB	5.01 ± 1.04	5.46 ± 0.39	12.00 ± 0.28	23.20 ± 2.69

### 2.4 PEI‐MSNs Loaded with Growth Factors Promote Functional Hepatic Maturation

**Figure 4:**
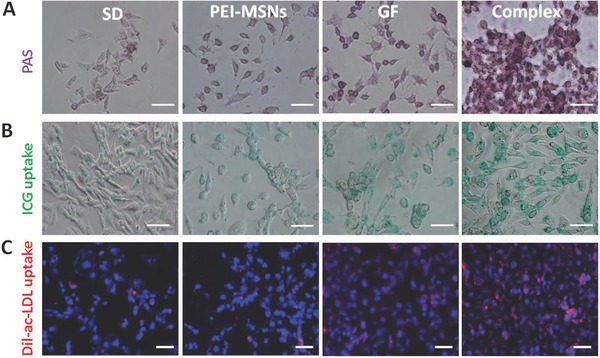
Functional tests of mouse embryonic stem cell (mESC)‐derived hepatocyte‐like cells (iHeps) induced by GF‐PEI‐MSN complexes, PEI‐MSNs, growth factors alone, and without treatment. A) The functions of glycogen synthesis and storage were measured by periodic acid‐Schiff (PAS) assays on the mESC‐derived cells in the four groups. Glycogen storage is indicated by pink or dark red‐purple cytoplasms. B) The cellular uptake function of indocyanine green (ICG), and C) low‐density lipoprotein (LDL) in the different treatment groups was analyzed using a fluorescence microscope at the end of the differentiation process. Scale bar: 50 μm.

We further examined whether PEI‐MSNs loaded with growth factor complexes can promote hepatic differentiation and attain mature liver function in the treated mESCs. Firstly, we tested the ability of the differentiating cell populations to store glycogen, which is an important function of mature hepatocytes, at day 18 using periodic acid‐Schiff (PAS) staining.^45^ A higher number of positive cells exhibited a pink to red‐purple cytoplasm in the GF‐PEI‐MSN complex treated group (**Figure** **4**A), whereas a small number of positive cells was detected in the different controls and treatments. In addition, the uptake of indocyanine green (ICG) and Dil‐labeled acetylated low density lipoprotein (Dil‐ac‐LDL) was investigated. ICG is a nontoxic organic anion that is eliminated exclusively by mature hepatocytes, the uptake, and release of ICG can be used to identify differentiated hepatocytes in vitro.^46^, ^47^ The differentiated cells displayed a pronounced capacity to take up ICG in the GF‐PEI‐MSN complex‐treated group (Figure 4B) compared with those of other groups. We also examined the capacity for Dil‐ac‐LDL uptake, which is a critical hepatocyte function.^48^ As shown in Figure 4C, the positive immunofluorescent signals for Dil‐ac‐LDL uptake in the GF‐PEI‐MSN complex group increased significantly in the hepatic lineage differentiated cells at day 18 compared with those of the control and other treatments. These results demonstrated that PEI‐MSNs delivering growth factors can promote efficient hepatic differentiation of mESCs and significantly improve the maturation of hepatocyte‐like cells with hepatic functionality.

### 2.5 Functional Evaluation of Transplantation of mESC‐Derived Definitive Endoderm Cells into Mice with CCl_4_ Injury

**Figure 5:**
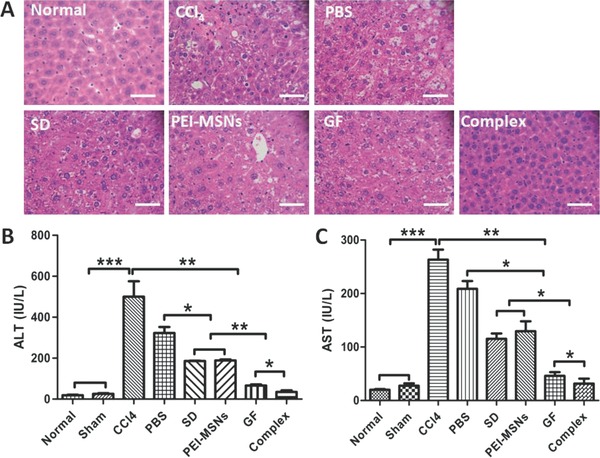
Evaluation of transplantation of mESC‐derived DE cells by GF‐PEI‐MSN complexes, PEI‐MSNs, growth factors alone, and without treatment at four weeks in the recovery of the CCl_4_‐injured mouse model. A) Hematoxylin and eosin (H&E) staining was performed on mouse liver sections in the different treatment groups. Scale bar: 50 μm. B) Serum ALT and C) AST levels in the different groups were measured by enzyme‐linked immunosorbent assays. All data are represented as the mean ± SD (*n* = 3). *P < 0.05, **P < 0.01, and ***P < 0.001. Abbreviations: Normal, untreated normal mice; Sham, CCl_4_ was not administered; CCl_4_, CCl_4_ group; PBS, PBS group.

To further test the function of transplanted mESC‐derived DE cells induced by PEI‐MSNs loaded with growth factors in vivo, we used a mouse transplantation model for hepatic repopulation following carbon tetrachloride (CCl_4_)‐induced liver injury (Figure S4, Supporting Information). Studies have reported that transplanted cells have a selective advantage over host hepatocytes in injured livers.^49^ Therefore, the four types of DE cells were derived from three‐day treatment with PEI‐MSNs, growth factors only and with GF‐PEI‐MSN complexes or without treatment and injected intrasplenically to mice with CCl_4_ Injury. After 2 d of cell transplantation, the livers were harvested, and sections were examined by hematoxylin and eosin (H&E) staining. All of the livers were characterized by acute focal necrosis, ballooning necrosis, steatosis, and inflammatory cell infiltration in the treated groups compared with the control group (Figure S5, Supporting Information). After four weeks, all of the cell transplantations had ameliorated the effects of chronic liver injury and fibrosis. In addition, the cell transplanted livers in the GF‐PEI‐MSN complex group showed improved liver architecture that was similar to normal mice liver and lessened or absent fibrosis compared with the other groups (**Figure** **5**A). Additionally, similar findings were also confirmed by enzyme‐linked immunosorbent assay (ELISA) analyses, which showed that serum alanine aminotransferase (ALT) (Figure 5B) and aspartate aminotransferase (AST) (Figure 5C) levels had decreased significantly with growth factors alone and the GF‐PEI‐MSN complex groups compared with the other groups. Moreover, the serum levels of ALT and AST returned to basal levels of normal mice or sham mice in the GF‐PEI‐MSN complex group. These findings support the conclusion that growth factors delivered by PEI‐MSNs improved the rescuing efficiency in CCl_4_‐injured liver.

To date, numerous studies in rodents have shown that predifferentiated ESC transplantation can reverse acute fulminant hepatic failure.^6^,^50^, ^51^, ^52^, ^53^, ^54^ In our study, we employed day‐3‐induced DE cells as a source of isolated cells for transplantation into injured liver. The results showed DE cells were able to differentiate into hepatocyte‐like cells and attenuate AST and ALT levels after transplantation into injured liver, which confirmed the results of related reports.^55^, ^56^, ^57^ To our knowledge, this is the first report demonstrating that PEI‐MSNs can be used as a delivery system for growth factors to improve DE cells from mESCs in vivo and significantly restore liver function. Although further studies will be necessary to determine the most suitable stages of mESCs differentiation procedure for transplantation, these results suggest the potential application of DE cells differentiated from mESCs for therapy of liver injuries or diseases.

### 2.6 Engraftment and Derivation of Transplanted Cells

**Figure 6:**
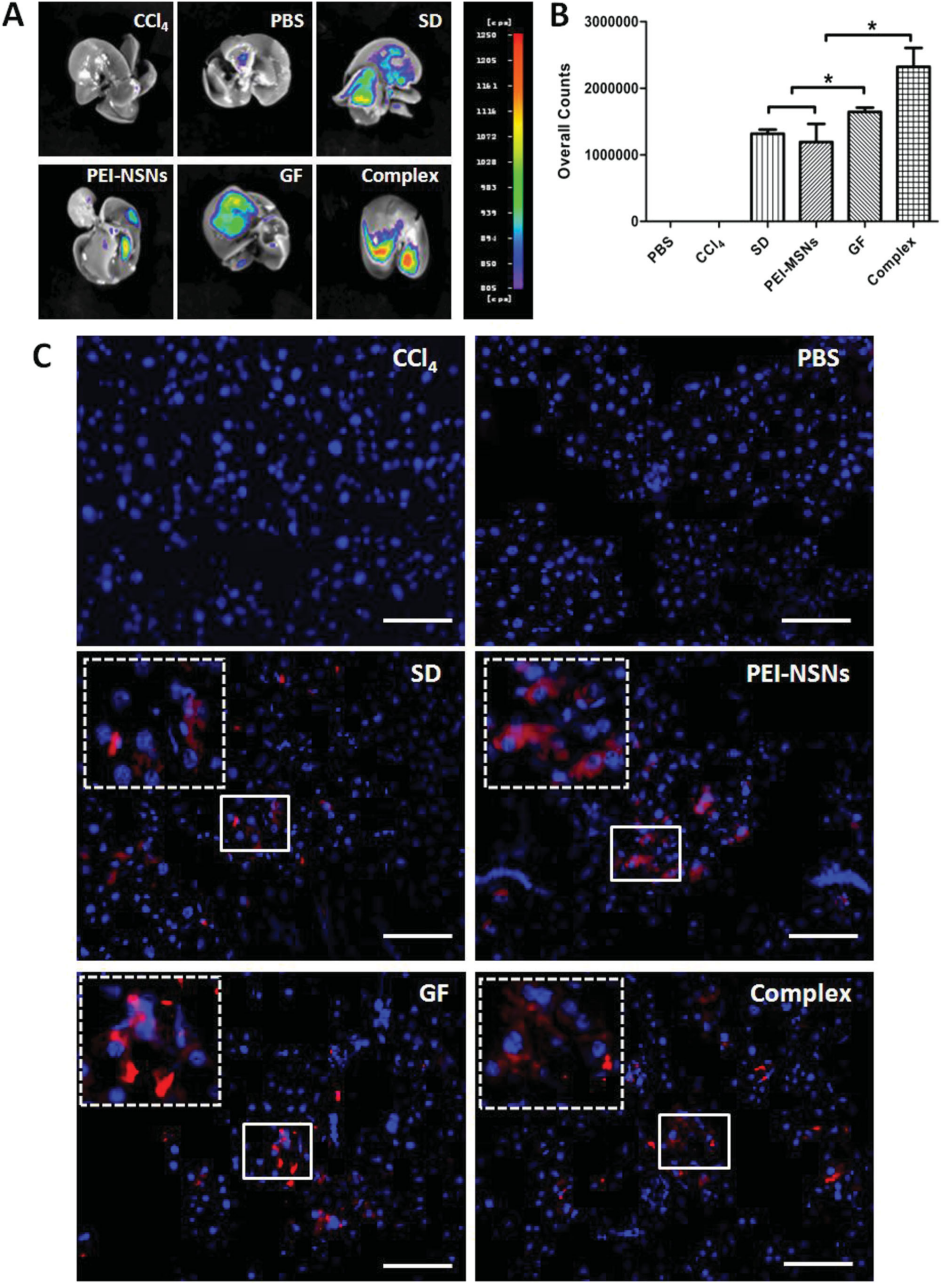
Cell detection of transplanted mESC‐derived DE cells from the different treatments in the CCl_4_ injured mouse model. A) Cells were labeled with CM‐Dil prior to transplantation, and after four weeks, the transplanted cells were detected in the whole mouse host liver using an in vivo bioimaging system. B) Quantitative analysis of the transplanted cells from the different treatments shown in A. All data are represented as the mean ± SD (*n* = 3), *P < 0.05. C) Representative fluorescence images of the liver sections at four weeks after transplantation of CM‐Dil labeled cells. Inset, high magnification images of CM‐Dil labeled cells. Scale bar: 100 μm. Red, CM‐Dil; blue, DAPI.

Efficient cell engraftment and retention is critical for successful cell‐based therapy. To trace the homing of transplanted cells, liver sections were examined by in vivo bioimaging (**Figure** **6**A,B) and immunofluorescence (Figure 6C). Four weeks after transplantation, the red fluorescent signal of CM‐Dil‐labeled DE cells (chloromethylbenzamido(Cell Tracker CM‐DiI))was still detected in the mice livers (Figure 6A). Furthermore, mESC‐derived DE cells treated with the GF‐PEI‐MSN complexes for 3 d exhibited a greater homing ability to detect in the injured liver compared with the other groups (Figure 6B). These results were further confirmed by the CM‐Dil‐labeled cells detected in the recipient livers (Figure 6C and single channel images shown in Figure S6 in the Supporting Information), which also revealed that more cells induced by GF‐PEI‐MSN complexes were engrafted into host livers.

**Figure 7:**
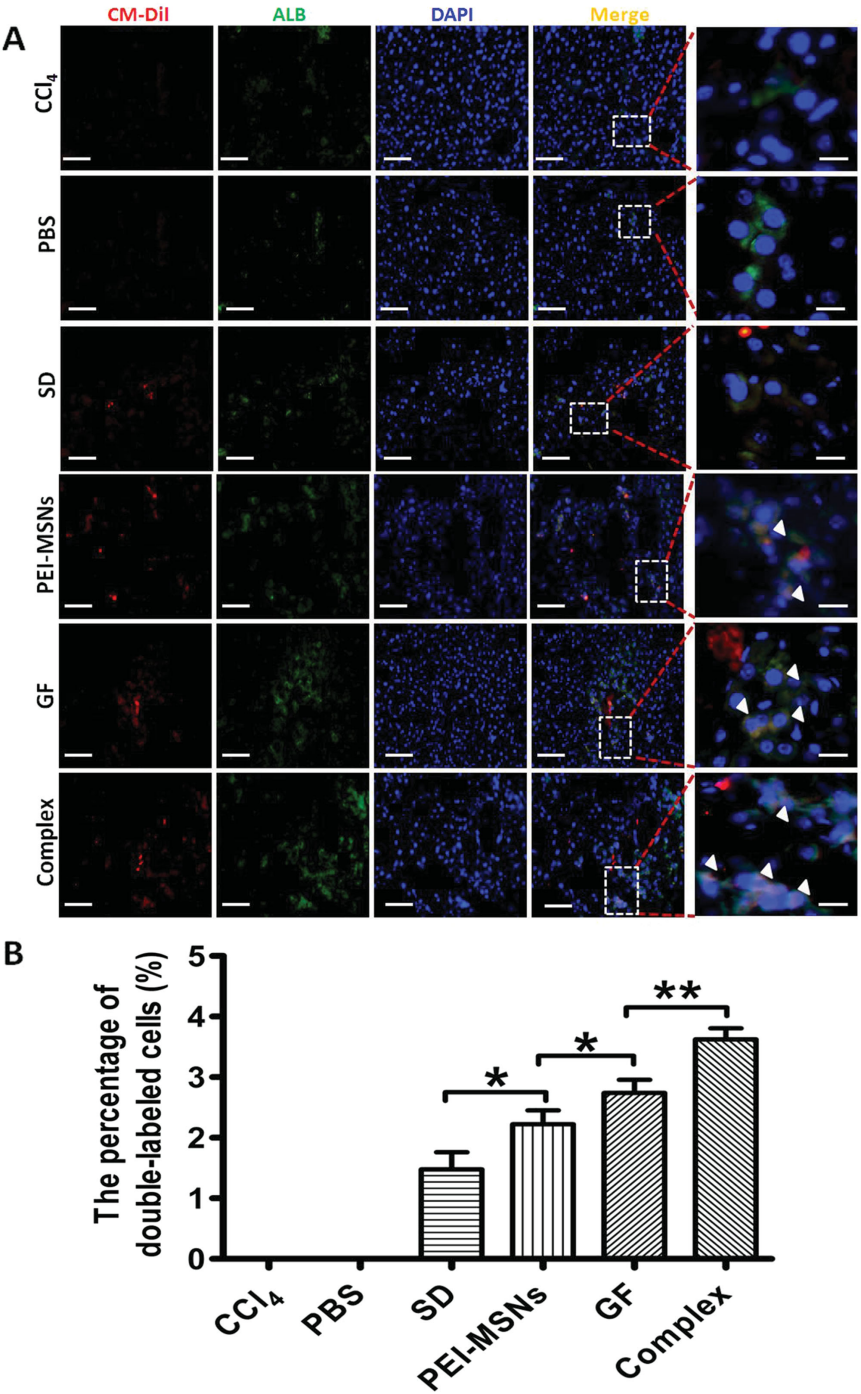
Cell differentiation of transplanted mESC‐derived DE cells from the different treatments in vivo for hepatic repopulation. A) The transplanted livers were detected by fluorescence microscopy staining in the different groups. The exogenous origin of the differentiated hepatocyte‐like cells was confirmed by costaining for CM‐Dil (red) and ALB (green). The nuclei were counterstained with DAPI (blue). Scale bar: 100 μm. Inset and white arrowhead, high magnification images of differentiated cells. Representative field showing no CM‐Dil staining in a nontransplanted liver section (CCl_4_ or PBS). B) Quantification of differentiated CM‐Dil+/ALB+ cells shown in A. All data are represented as the mean ± SD (*n* = 3). *P < 0.05 and **P < 0.01.

To evaluate hepatocyte differentiation of engrafted cells in the livers, ALB was used as a marker of mature hepatocytes, and the transplanted cells were evaluated by immunofluorescence staining. Although a small number of double CM‐Dil^+^/ALB^+^ cells were detected in the livers of all recipients (**Figure** **7**A), there was a higher percentage of double CM‐Dil^+^/ALB^+^ cells (3.62% ± 0.19%) in the GF‐PEI‐MSN complex group compared with the growth factors (2.74% ± 0.22%), PEI‐MSNs (2.22% ± 0.23%), and control groups (1.48% ± 0.28%) (Figure 7B). Moreover, under our conditions, teratoma formation (Figure S7, Supporting Information) was not observed in a series of grafts, although further studies using different animal models of liver diseases are needed to address the long‐term safety and efficacy of DE cells. These findings demonstrate that PEI‐MSNs carrying growth factors can act as an efficient platform to improve engraftment of transplanted DE cells to an injured liver and promote differentiation into hepatocyte‐like cells in vivo compared with controls and other treatments.

Two primary mechanisms may explain the therapeutic effects of transplanted cells in injured liver.^58^ First, ESCs could generate functional hepatocytes in vivo that are then engrafted efficiently within the host liver.^6^ A study reported that 2.5%–5% is necessary for reversing the liver injuries.^59^ We also observed additional CM‐Dil^+^/ALB^+^ cells in the GF‐PEI‐MSN complex group; however, low amounts of these cells could not significantly reverse the injured liver, which was mentioned by previous investigators.^60^ One explanation for this low differentiation level may be that limited space is available for the donor cells to integrate into the CCl_4_‐treated mouse,^61^ and they do so with low efficiency. The second mechanism of promoting liver repair is indirect. Secretomes released from stem cells or their derivatives contribute to liver regeneration in response to acute damage.^62^ Our study showed that a few engrafted cells expressed ALB in recipient livers, whereas these cells can improve liver function in a mouse disease model, which may also be related to the paracrine effect of predifferentiated cells.

## 3 Conclusions

Stem‐cell‐derived hepatocyte transplantation is currently being evaluated as a potential method of providing metabolic support during acute and chronic liver failure, but is restricted due to inefficient differentiation and low purity of functional hepatocytes. In this study, we have successfully established a polymer‐modified nanoparticles‐based sustained delivery system for growth factors to direct stem cell differentiated into hepatocytes. Our findings show that this approach can help to overcome the limitations associated with current models and ensure efficient delivery of growth factors to improve mESC differentiation toward a hepatocyte‐like lineage with mature liver functions in vitro, including glycogen storage, indocyanine green and low‐density lipoprotein uptake. When transplanted into mice with liver injury, this system significantly repopulated the damaged liver, which was attributed to transplanted cells and the paracrine effect of predifferentiated cells. Therefore, MSNs with multifunctional surface properties are suitable delivery platforms for biomolecule delivery to induce specific differentiation and cell‐based therapies for treatment of hepatic disease and tissue engineering, which provides a powerful system not only for efficient differentiation of the stem cell, but also for developing therapeutic strategies in regenerative medicine.

## 4 Experimental Section

*Materials*: Tetraethylorthosilicate (TEOS) and PEI (25 kD) were purchased from Sigma‐Aldrich (St. Louis, MO, USA). Cetyltrimethylammonium bromide (CTAB) was purchased from Alfa Aesar (Tianjin, China). All tissue culture materials were obtained from Gibco (Grand Island, NY, USA), and the other reagents (analytical grade) were purchased from Sigma‐Aldrich. All of the chemicals were of analytical grade and used without further purification.

*Animals*: Experiments were performed using male ICR mice weighing 20–25 g and aged six to eight weeks (Laboratory Animal Center of Tongji University). All of the animal research procedures were approved by the Animal Experimentation Committee of Tongji University, and the animals were cared for in accordance with the Guidelines for Animal Experiments of Tongji University.

*Synthesis and Surface Modification of Mesoporous Silica Nanoparticles*: The synthesis of MSNs was performed according to an earlier publication.^30^, ^31^ Briefly, 300 mg CTAB was dissolved in a solution of 145 mL distilled water and 1.05 mL sodium hydroxide (2.0 m). The solution was heated to 80 °C, and then TEOS (2.5 mL) was added. The solution was vigorously stirred, and the reaction mixture was then stirred for 2 h. The resulting suspension was centrifuged and washed with water and ethanol several times. The precipitate was dried at 60 °C. To remove the surfactant template (CTAB), the samples were calcined for 5 h at 550 °C.

PEI was coated onto the MSNs according to procedures described in an earlier publication.^19^ MSNs (10 mg) were added to a solution containing 5 mg PEI (MW 25 kD) and 1 mL absolute ethanol. After the mixture was sonicated and stirred for 30 min, PEI‐coated MSNs were washed with ethanol and water.

*Characterization*: The shapes and structures of the MSNs were characterized using a TEM (JEM 2011, JEOL, Japan). The particle sizes and *ζ* potentials were determined in PBS using a Malvern Zetasizer (Nano Series, Malvern Instruments Inc., MA, USA). FT‐IR absorption spectra were recorded on a Nicolet Nexus 470 spectrometer. The total amount of PEI coating was determined by TGA (TGA7, PerkinElmer, USA). The MSN pore size, volume, and specific surface area were determined by nitrogen sorption measurements (Micromeritics TriStar 3000 analyzer, Micromeritics, USA). The surface areas and pore size distributions of the samples were calculated by the BET and Barrett−Joyner−Halenda (BJH) methods, respectively.

*Growth Factor Loading and Release*: PEI‐coated MSNs were loaded with Activin A (R&D Systems, Minneapolis, MN, USA), aFGF (PeproTech, Rocky Hill, NJ, USA), and HGF (R&D Systems) by incubating 10 mg nanoparticles (sterilized under UV light) in a solution of 12 μg mL^−1^ Activin A, 5 mg sterilized nanoparticles in a solution of 1.8 μg mL^−1^ aFGF, and 5 mg sterilized nanoparticles in a solution of 1.3 μg mL^−1^ HGF, respectively, for 24 h at 4 °C in a thermomixer comfort (Eppendorf, Hamburg, Germany) with constant shaking at 1000 rpm. Following repetitive washings of the growth‐factor‐laden nanoparticles and removal of unloaded growth factors by centrifugation, the precipitates, Activin A‐PEI‐MSNs, aFGF‐PEI‐MSNs, and HGF‐PEI‐MSNs were redispersed in PBS for use in future experiments. The free growth factors present in the supernatant were determined using an ELISA according to the manufacturer's instructions. The growth factor loading efficiency of nanoparticles was calculated using the following equation

$$\mathrm{Loading}\;\mathrm{efficiency}=\;\frac{\left(\mathrm{total}\;\mathrm{growth}\;\mathrm{factor}-\mathrm{free}\;\mathrm{growth}\;\mathrm{factor}\right)}{\mathrm{total}\;\mathrm{growth}\;\mathrm{factor}}$$

In the release experiments, the growth‐factor‐laden nanoparticles prepared were placed in PBS (5 mL) and incubated under mild agitation at 37 °C. At predetermined time intervals, the particle suspension was centrifuged (at 10 000 rpm for 5 min) and the supernatant (4 mL) was removed and replaced by a new one. The amount of growth factors released was quantified by ELISA (R&D Systems). All analyses were conducted in duplicate.

*Mouse Embryonic Stem Cell Cultures*: Undifferentiated mESC D3 cells were obtained from Professor Xiaoqing Zhang, School of Medicine, Tongji University. mESC D3 cells were maintained on irradiated mouse embryonic fibroblast feeder cells in 0.1% gelatin‐coated dishes in Dulbecco's modified Eagle's medium (DMEM) supplemented with 15% (V/V) fetal bovine serum (FBS), 1% (V/V) non‐essential amino acids, 1 × 10^−3^ m GlutaMAX, 0.1 × 10^−3^ m β‐mercaptoethanol (all from Gibco), and 1000 U mL^−1^ recombinant mouse leukemia inhibitory factor (LIF; Millipore, CA, USA). Media were changed every day.

*Hepatic Differentiation In Vitro*: To evaluate the effect of GF‐PEI‐MSN complexes on hepatocyte differentiation, the mESCs were cultured according to previously published protocols with minor modifications as described in Figure 2A. Before the initiation of cellular differentiation, the mESCs were dissociated into single cells at a seeding density of 1 × 10^5^ cells mL^−1^ and cultured in 12‐well tissue culture plates (Corning, NY, USA) coated with 0.1% gelatin (Sigma‐Aldrich) and without a feeder layer. To direct the differentiation of mESCs into hepatocytes, differentiation was induced by treating mESCs with differentiation medium that consisted of Glasgow minimum essential medium (GMEM, Gibco) supplemented with 2% FBS and Activin A‐PEI‐MSN complexes (100 μg mL^−1^ PEI‐MSNs and 100 ng mL^−1^ Activin A) for 3 d followed by treatment with differentiation medium consisting of GMEM supplemented with 10% FBS, 2.5 × 10^−3^ m sodium butyrate (Sigma‐Aldrich) and aFGF‐PEI‐MSN complexes (100 μg mL^−1^ PEI‐MSNs and 30 ng mL^−1^) for 5 d. The cells were further cultured in the maturation medium, which consisted of GMEM media supplemented with HGF‐PEI‐MSN complexes (100 μg mL^−1^ PEI‐MSNs and 20 ng mL^−1^ HGF) for 5 d and then followed by 5 d in 10 ng mL^−1^ oncostatin M (OSM; R&D Systems) plus 0.1 × 10^−3^ m dexamethasone (Dex; Sigma‐Aldrich). Experimental groups included mESCs with PEI‐MSNs loaded with Activin A, aFGF or HGF, whereas control groups included mESCs cells with or without an equal quantity PEI‐MSNs and growth factors only.

*RNA Extraction and Quantitative Reverse Transcriptase Polymerase Chain Reaction*: Total RNA was extracted from cells on different differentiation days using RNAiso Plus (TaKaRa Bio Inc, Japan) and treated with Recombinant DNase I (RNase‐free) (TaKaRa Bio Inc, Japan) to remove genomic DNA contamination following the manufacturer's protocol. A total of 1 μg RNA was reverse transcribed into cDNA in a volume of 20 μL with M‐MLV ReverseTranscriptase (Promega, WI, USA) according to the manufacturer's instructions. qPCR analysis was performed on a BioRad iQ5 Real‐Time PCR System using the SYBR Green qPCR Master Mix (Bio‐Rad, California, USA). The PCR reaction consisted of 10 μL 2× SYBR Green PCR Master Mix, 1 μL 5 × 10^−6^ m forward and reverse primers, 8 μL water, and 1.0 μL template cDNA in a total volume of 20 μL. Conditions for PCR amplifications were as follows: 95 °C for 5 min, 40 thermal cycles of 95 °C for 30 s, 60 °C for 30 s, 72 °C for 30 s, and a final extension at 72 °C for 10 min. The specific primers used for the qPCR are listed in Table S3(Supporting Information). Each qPCR quantification experiment was performed in triplicate for each individual sample. The final results were reported as the relative expression normalized with the transcript level of the housekeeping gene glyceraldehyde 3‐phosphate dehydrogenase (GAPDH) using the comparative CT Method (2^−CT^).

*Immunofluorescence Staining*: Cells were fixed for 20 min at 4 °C in 4% paraformaldehyde and then washed three times in PBS. Cells were permeabilized in 0.2% Triton X‐100 and then blocked with 10% goat serum in PBS for 20 min before incubation overnight at 4 °C with primary antibody diluted in 1% goat serum in PBS as follows: mouse anti‐Sox17 (1:100, R&D Systems), rabbit anti‐FoxA2 (1:500, Millipore), mouse anti‐CK18 (1:100, Abcam), goat anti‐AFP (1:50, Santa Cruz), and sheep anti‐ALB (1:100, Abcam). The cells were then washed three times in PBS and incubated with secondary antibodies, including rabbit anti‐mouse Alexa Fluor 488 conjugated (1:100), goat anti‐rabbit PE‐conjugated (1:200), rabbit anti‐mouse PE conjugated (1:200), rabbit anti‐goat fluorescein isothiocyanate conjugated (1:100), or rabbit anti‐sheep fluorescein isothiocyanate conjugated (1:100) (Jackson ImmunoResearch) for 1 h at room temperature. The cells were washed three times for 5 min each with PBS, counterstained with 4,6‐diamidino‐2‐phenylindole (DAPI) (0.25 μg mL^−1^, Molecular Probes) for 10 min, rinsed with PBS three times for 15 min and observed using an inverted fluorescence microscope (Leica DMI 4000B, Heerbrugg, Switzerland).

*Flow Cytometry*: The cells were harvested by digestion with 0.125% trypsin/ethylenediaminetetraacetic acid (EDTA), fixed with 4% paraformaldehyde for 30 min, and then permeabilized in staining buffer (PBS with 10% bovine serum albumin (BSA, Sigma‐Aldrich) and 0.2% Triton X‐100) for 10 min. The cells were then incubated for 1 h with primary antibody against Sox17 (1:20, R&D Systems), FoxA2 (1:50, Millipore), AFP (1:200, Santa Cruz), or ALB (1:20, Abcam). After washing, the cells were incubated for 30 min with Alexa Fluor 488‐conjugated anti‐rabbit or anti‐mouse secondary antibody (Jackson ImmunoResearch) diluted to 1:100. Afterward, the cells were washed three times with PBS buffer. Finally, the cells were resuspended in 0.5 mL ice‐cold PBS and analyzed using a Calibur flow cytometer (Becton Dickinson, CA, USA). Data were analyzed with the software FlowJo (Tree Star, version 7.6).

*Periodic Acid‐Schiff Staining*: The PAS staining system was purchased from Sigma‐Aldrich. Culture dishes containing cells were fixed in 4% paraformaldehyde, and the assay was performed according to the manufacturer's instructions.

*Indocyanine Green and Low‐Density Lipoprotein Uptake*: For ICG (Sigma‐Aldrich) uptake assay, ICG was suspended in dimethyl sulfoxide (DMSO) (Sigma‐Aldrich) and a stock solution at 5 mg mL^−1^ and freshly diluted in culture medium to a final concentration of 1 mg mL^−1^. The cells were incubated in diluted ICG for 30 min at 37 °C followed by washing with PBS three times. The cells were returned to the culture medium, and the release of ICG was evaluated 6 h later.

For the LDL uptake test, the differentiated cells were incubated in DMEM containing 10 μg mL^−1^ acetylated low‐density lipoprotein labeled with 1,1′‐dioctadecyl‐3,3,3′3 ′‐tetramethylindocarbocyanine perchlorate (Dil‐Ac‐LDL, Invitrogen, USA) for 4 h at 37 °C. The cells were washed three times for 5 min each with PBS, counterstained with DAPI for 10 min, rinsed with PBS three times, and visualized using an inverted fluorescence microscope (Leica DMI 4000B).

*Animal Treatment and Cell Transplantation*: To induce liver fibrosis, each mouse was administered intragastrically 1 mL kg^−1^ body weight 20% (V/V) CCl_4_ (Chemical Reagent Company, Shanghai, China) dissolved in corn oil (Alfa Aesar, Tianjin, China) three times per week for four weeks. The day‐3 differentiated cells from different treatments were trypsinized at 37 °C with 0.125% trypsin/EDTA and then resuspended in PBS. Prior to implantation, the cells were labeled with CM‐Dil (Life Technologies) according to the manufacturer's recommendations. Approximately 1 × 10^6^ cells in 0.1 mL suspension were injected intrasplenically into ICR mice (*n* = 5). In the normal (*n* = 5) groups and sham treatment (*n* = 5) groups, CCl_4_ was not administered. In the sham group, the mice were injected with the vehicle (corn oil) alone (three times per week for eight weeks). In the CCl_4_ group, ICR mice were randomly divided into six groups: (1) mice injected with CCl_4_ alone without cells; (2) mice injected with 0.1 mL PBS (used as negative controls); (3) mice administered spontaneously differentiated mESCs cultured in the absence of PEI‐MSNs or any growth factors; (4) mice administered differentiated mESCs treated with PEI‐MSNs alone; (5) mice administered differentiated mESCs treated with growth factors alone; and (6) mice administered differentiated mESCs treated with GF‐PEI‐MSN complexes. Each experimental group contained five mice. Histological analysis of liver tissues was conducted by serial tissue section at two days and four weeks after cell transplantation. The blood at four weeks was harvested for further analysis. To observe the fate of transplanted cells in mice with liver injury, in vivo imaging was performed using a NightOWL imaging system and WinLight software (Berthold Technologies, Germany).

*Histology and Immunofluorescence*: The livers were fixed in PBS containing 4% formaldehyde, embedded in paraffin, and sectioned into 8 μm sections. Sample sections were stained with H&E. For immunofluorescence staining, the livers were fixed and embedded in OCT compound (Tissue‐TEK, Sakura Finetek, CA, USA). After fixation, cryosections (8 μm) were incubated with ALB primary antibodies (1:100, Abcam) at 4 °C overnight, and the sections were then incubated with secondary antibodies at room temperature for 1 h. Finally, samples were counterstained with DAPI for 10 min. Sample sections were visualized and imaged using a fluorescence microscope.

*Aminotransferase Analysis*: Blood samples were centrifuged at 3000 ×*g* for 10 min to separate the serum, and they were then stored at −80 °C for subsequent analyses. ALT and AST were determined using commercial enzymatic kits (Jiancheng, Nanjing, China).

*Statistical Analysis*: The results are given as the mean ± SD. An unpaired *t*‐test or one‐way analysis of variance with Bonferroni post‐test was performed with the software GraphPad Prism 5.0 (San Diego, CA, USA). The results were considered significant at *P* < 0.05 (*), very significant at *P* < 0.01 (**), and extremely significant at *P* < 0.001 (***).

## Supporting information

As a service to our authors and readers, this journal provides supporting information supplied by the authors. Such materials are peer reviewed and may be re‐organized for online delivery, but are not copy‐edited or typeset. Technical support issues arising from supporting information (other than missing files) should be addressed to the authors.

Supplementary
