# Supplementary material for: Sustained Delivery Growth Factors with Polyethyleneimine‐Modified Nanoparticles Promote Embryonic Stem Cells Differentiation and Liver Regeneration
Source: Adv Sci (Weinh). 2016 Apr 8;3(8):1500393. doi: 10.1002/advs.201500393 (PMC5071678; doi:10.1002/advs.201500393)
Supplement: Supplementary file 1 — Supplementary [file ADVS-3-0b-s001.pdf]

Copyright WILEY-VCH Verlag GmbH & Co. KGaA, 69469 Weinheim, Germany, 2016.

ADVANCED SCIENCE
Open Access

Supporting Information

for *Adv. Sci.*, DOI: 10.1002/advs.201500393

Sustained Delivery Growth Factors with Polyethyleneimine-Modified Nanoparticles Promote Embryonic Stem Cells Differentiation and Liver Regeneration

Meiyan Wang, Xiaomei Yang, Peng Zhang, Lei Cai, Xibin Yang, Youwei Chen, Yuanya Jing, Jilie Kong, Xiaowei Yang, and Fang-Lin Sun**

Copyright WILEY-VCH Verlag GmbH & Co. KGaA, 69469 Weinheim, Germany, 2015.

Supporting Information

Sustained Delivering Growth Factors with Polyethyleneimine Modified Nanoparticles Promotes Embryonic Stem Cells Differentiation and Liver Regeneration

Meiyan Wang, Peng Zhang, Lei Cai, Xibin Yang, Youwei Chen, Yuanya Jing , Xiaomei Yang , Jilie Kong, Xiaowei Yang, and Fang-Lin Sun**

Table of contents:

Figure S1 Percentage cell survival of mESC cells upon exposure to different MSNs or PEI-MSNs for 24 h and 48 h, respectively assessed using an CCK8 cell viability kit. Data are presented as means ± SD of six independent experiments.

Figure S2 Characterization of mESCs-derived Definitive endoderm (DE) cells and hepatocyte-like cells (iHeps) by GF-PEI-MSN complexes, PEI-MSNs, growth factors alone, and without treatment *in vitro* at day 3, 18. The derived cells were stained with antibodies against Definitive endoderm (DE) cells markers Sox17(A) and FoxA2(B) and liver-specific markers CK18(C), AFP(D) and ALB(E), Nuclei were counterstained with DAPI (4',6-diamidino-2-phenylindole). From left to right: bright field images, nucleus staining fluorescence images, fluorescence images. Scale bar = 200 μm.

Figure S3 Flow cytometry profiles of mESC-derived cells by GF-PEI-MSN complexes, PEI-MSNs, growth factors alone, and without treatment. The number of positively labeled cells is presented as the percentage of total counting cells in each condition.

Figure S4 Representative pictures of whole livers from normal mice and CCl_4-administered mice.

Figure S5 Microscopic analysis of representative histologic sections derived from CCl_4-treated mice liver tissue stained with hematoxylin and eosin (H&E) at 2 day after the transplantation of mESCs–derived DE cells by different treatment. Extensive liver damage areas (black arrow) were still observed in the different treatment groups. Scale bar = 100 μm.

Figure S6 Cell homing of transplanted mESC-derived DE cells from the different treatment in the CCl_4 injured mouse model. Representative fluorescence images of the liver sections at four weeks after transplantation of CM-Dil labeled cells with treatment of GF-PEI-MSN complexes, PEI- MSNs, growth factors only or without treatment. Cells were labeled with CM-Dil (red) prior to transplantation and the nuclei were counterstained with DAPI (blue), Scale bar = 100 μm.

Figure S7 Absence of teratoma formation. Teratoma was not observed in the transplantation of mESCs-derived DE cells by different treatment into mice with CCl_4-induced cirrhosis.

Table S1 Characterization of MSNs and modified MSNs

Table S2 Specific surface area (a_s,BET), total pore volume, and average pore diameters for MSNs and PEI-MSNs

Table S3 The sequences for primers of Q-PCR

Materials and Methods

Cellular Cytotoxicity: Cellular viability of MSNs or PEI-MSNs against mouse embryonic stem cells (mESCs) was evaluated by the CCK-8 assay (Beyotime, Beijing, China). The cells were plated onto 96-multi-well plates (Corning) previously coated with 0.1% gelatin (Sigma-Aldrich) and irradiated mouse embryonic fibroblastsd (MEF) feeder cells at a seeding density of 1 $\times 10^4$ cells per well and allowed to adhere overnight. The medium was changed and replaced with fresh medium containing different concentrations of MSNs or PEI-MSNs (*i.e.* 0, 15.625, 31.25, 62.5, 125 and 500 μg/mL, respectively) for 24 and 48 h at 37 ℃. After incubation, the medium was removed and the treated cells were washed twice with fresh DMEM. Cells in 96-well plate were added 100 μL of DMEM containing 10% CCK-8 solution and incubated another 4 h at 37 ℃. The absorbance at 450 nm of each well was measured using an automated ELISA reader (Bio-Tech Instruments, USA). The control samples were the mESCs culture media without adding MSNs or PEI-MSNs and the culture medium with premixed CCK-8 reagent was defined as a background control.

Results and Discussion

1. Biocompatibility of MSNs or PEI-MSNs

To assess cellular of MSNs or PEI-MSNs against mouse embryonic stem cells (mESCs), Cell viability and proliferation was assessed using CCK-8 assay following exposure to different concentrations of MSNs or PEI-MSNs. As shown in Figure S1, No obvious cytotoxicity was observed even at a high concentration (500 μg/mL) after 24 or 48 h incubation, which was similarly observed for MSNs or PEI-MSNs by other groups.[1-5] This

result indicates that the synthesized MSNs or PEI-MSNs were biocompatible and suitable as delivery vehicles.

2. Quantfication of growth factors loading

The selection of growth factors used is thought to play a crucial role in hepatic differentiation or development. It has been shown that differentiation medium are supplemented with 25-100 ng/ml Activin A, 20-30 ng/ml aFGF or 10-20ng/ml HGF at the different stages proved to be critical for the generation of hepatocytes from mESCs.[6-10] Firstly, from a pilot test, the growth factors (Activin A, aFGF and HGF) loading capacity of the nanoparticles was determined. This optimization method was performed three times. According to the loading efficiency, the calculated quantity of the growth factors used in the final loading study was added to the nanoparticles. The amount of growth factors (Activin A, aFGF and HGF) loading on those nanoparticles was determined by ELISA. For Activin A-PEI-MSNs, aFGF-PEI-MSNs and HGF-PEI-MSNs, It can be estimated that the PEI-MSNs bind about 82.23±7.98% Activin A , 81.30±0.18% aFGF or 77.91±4.57% HGF available in the incubation solution. In the present study, PEI-MSNs were applied at a concentration of 100 μg/mL PEI-MSNs. For Activin A, aFGF or HGF-loaded PEI-MSNs, this resulted in a concentration of about 100 ng/mL Activin A, 30 ng/mL aFGF or 20 ng/ml HGF in the differentiation medium.

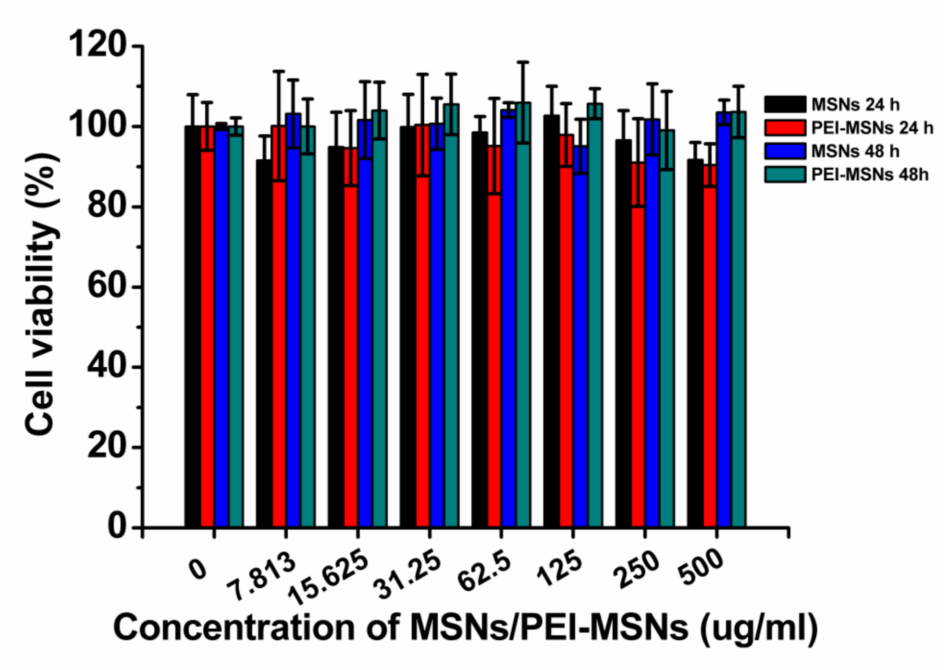

Figure S1 Percentage cell survival of mESC cells upon exposure to different MSNs or PEI-MSNs for 24 h and 48 h, respectively assessed using an CCK8 cell viability kit. Data are presented as means ±SD of six independent experiments.

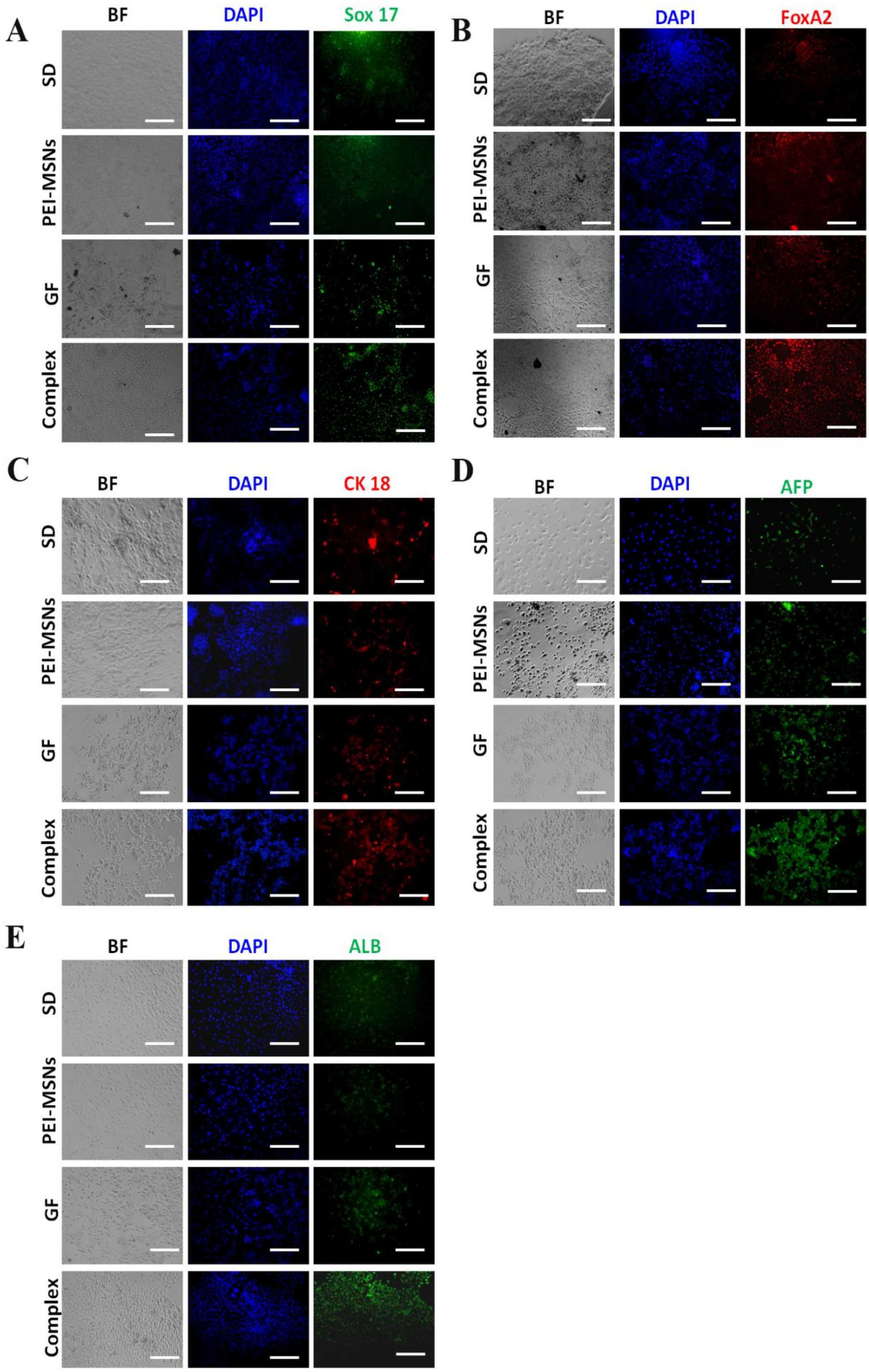
A
BF
DAPI
Sox 17
SD
PEI-MSNs
GF
Complex
B
BF
DAPI
FoxA2
SD
PEI-MSNs
GF
Complex
C
BF
DAPI
CK 18
SD
PEI-MSNs
GF
Complex
D
BF
DAPI
AFP
SD
PEI-MSNs
GF
Complex
E
BF
DAPI
ALB
SD
PEI-MSNs
GF
Complex

Figure S2 Characterization of mESCs-derived Definitive endoderm (DE) cells and hepatocyte-like cells (iHeps) by GF-PEI-MSN complexes, PEI-MSNs, growth factors alone, and without treatment *in vitro* at day 3, 18. The derived cells were stained with antibodies against Definitive endoderm (DE) cells markers Sox17(A) and FoxA2(B) and liver-specific markers CK18(C), AFP(D) and ALB(E), Nuclei were counterstained with DAPI (4',6-diamidino-2-phenylindole). From left to right: bright field images, nucleus staining fluorescence images, fluorescence images. Scale bar = 200 μm.

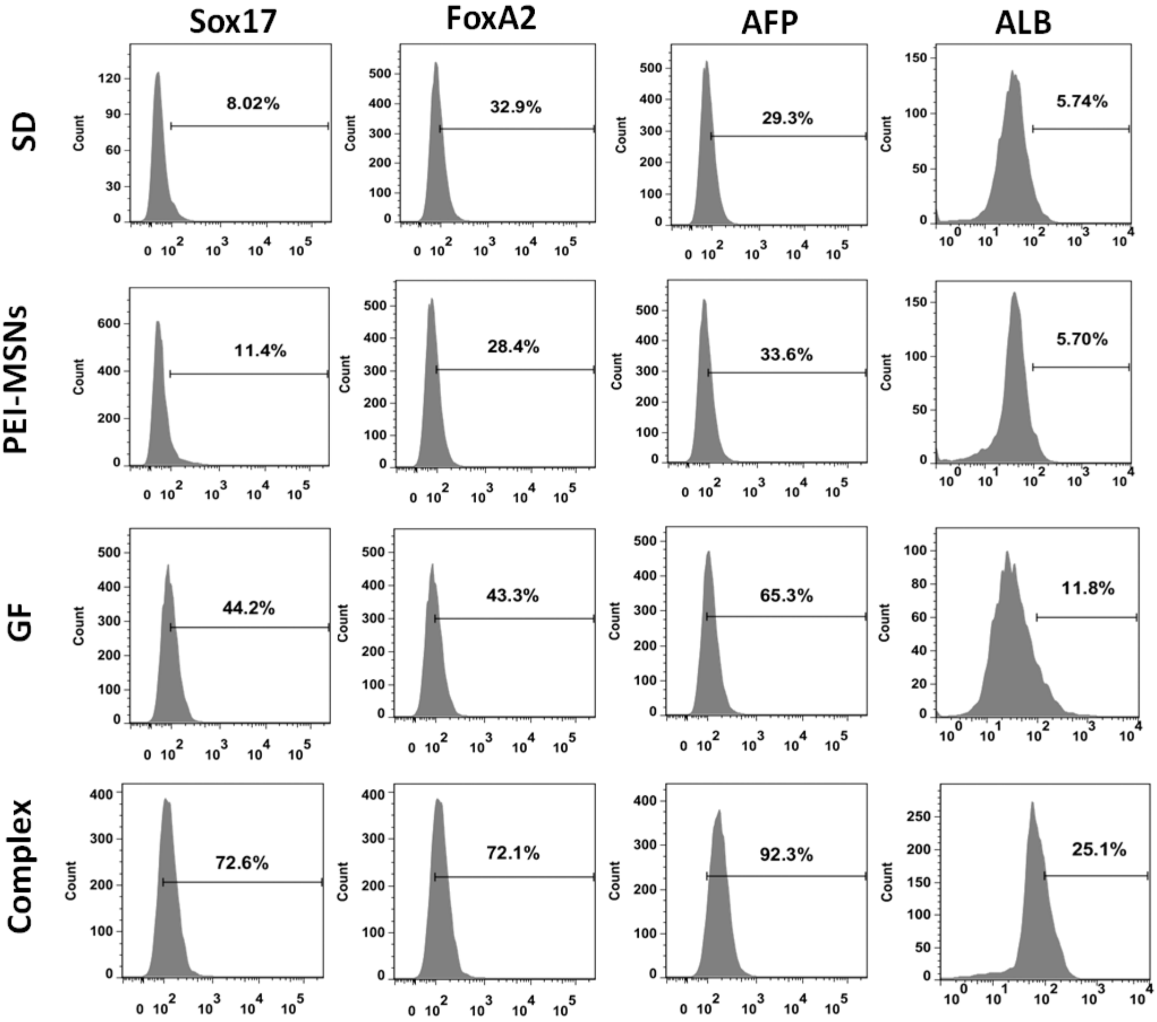

Figure S3 Flow cytometry profiles of mESC-derived cells by GF-PEI-MSN complexes, PEI-MSNs, growth factors alone, and without treatment. The number of positively labeled cells is presented as the percentage of total counting cells in each condition.

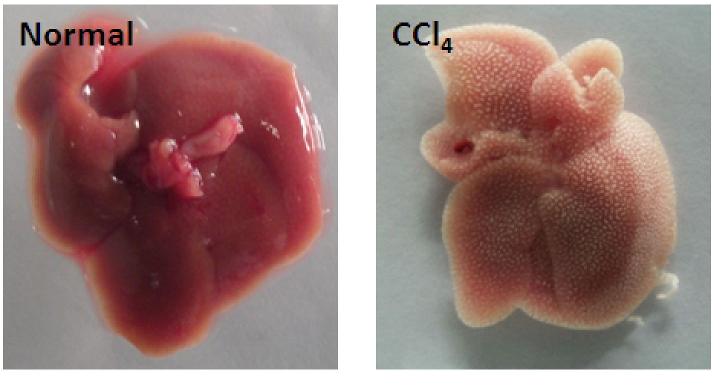

Figure S4 Representative pictures of whole livers from normal mice and CCl_4-administered mice.

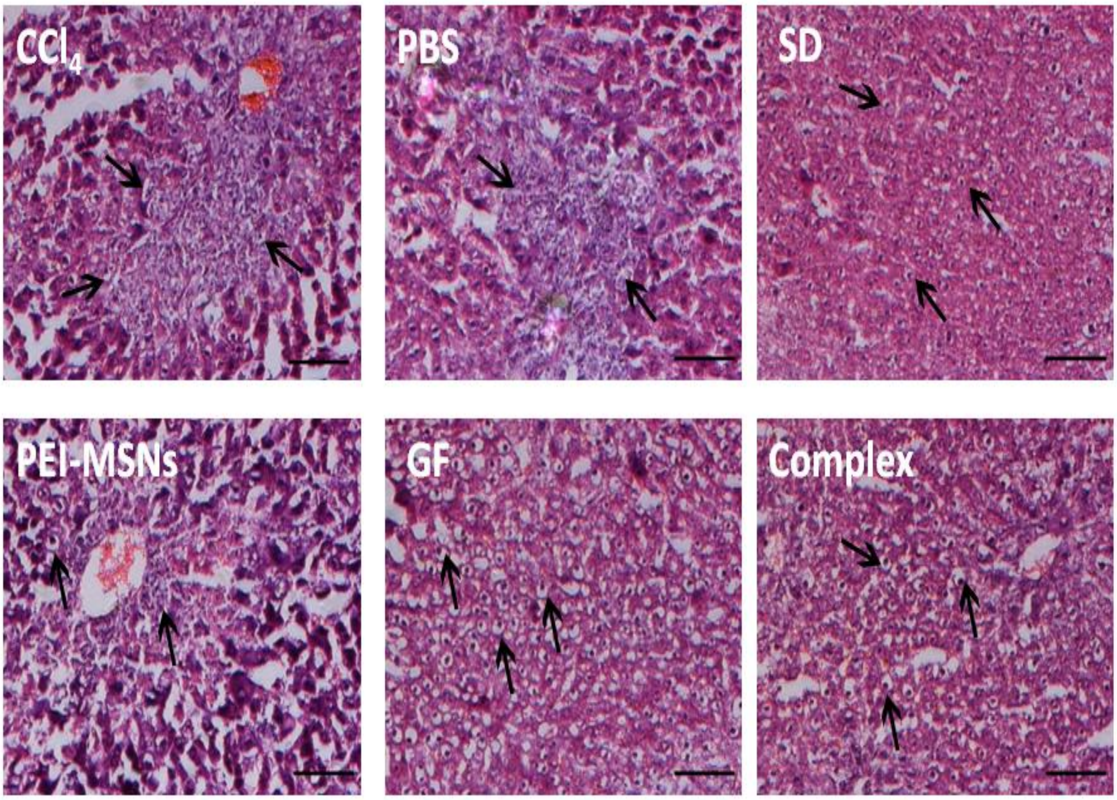

Figure S5 Microscopic analysis of representative histologic sections derived from CCl_4-treated mice liver tissue stained with hematoxylin and eosin (H&E) at 2 day after the transplantation of mESCs–derived DE cells by different treatment. Extensive liver damage areas (black arrow) were still observed in the different treatment groups. Scale bar = 100 μm.

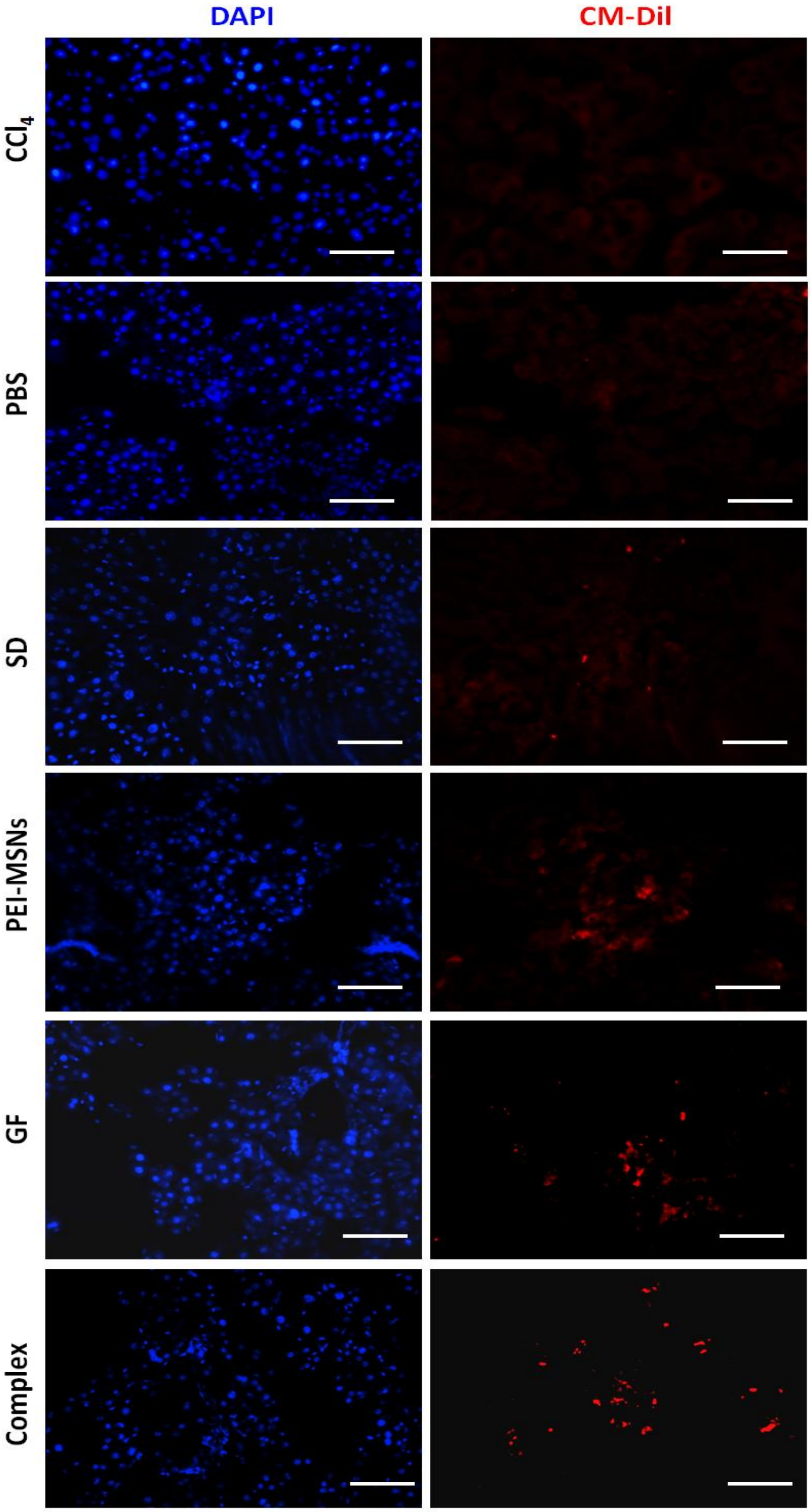
DAPI
CM-Dil
CCl_4
PBS
SD
PEI-MSNs
GF
Complex

Figure S6 Cell homing of transplanted mESC-derived DE cells from the different treatment in the CCl_4 injured mouse model. Representative fluorescence images of the liver sections at four weeks after transplantation of CM-Dil labeled cells with treatment of GF-PEI-MSN complexes, PEI- MSNs, growth factors only or without treatment. Cells were labeled with CM-Dil (red) prior to transplantation and the nuclei were counterstained with DAPI (blue), Scale bar = 100 μm.

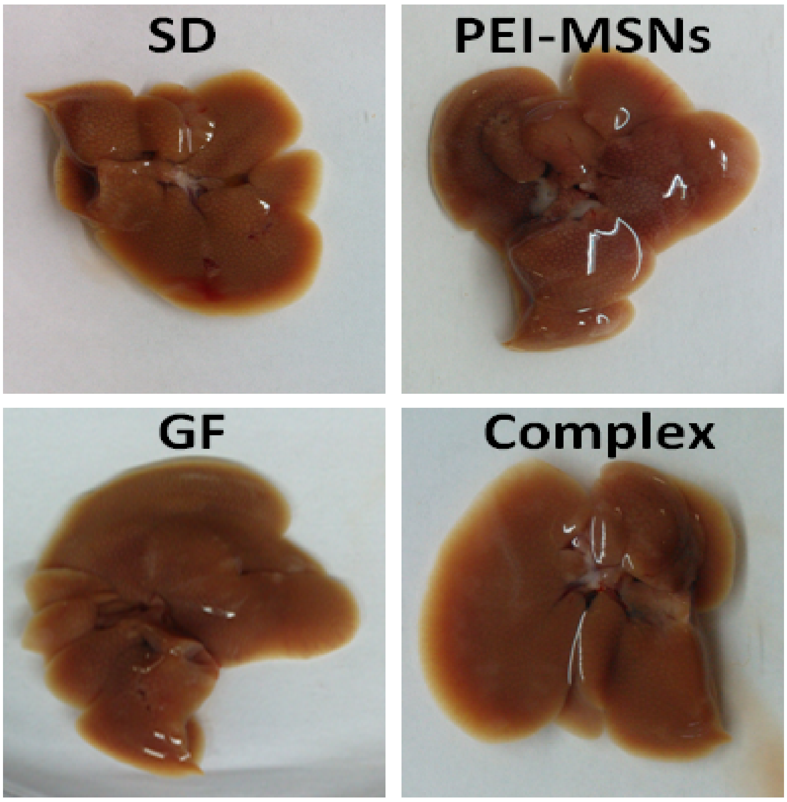

Figure S7 Absence of teratoma formation. Teratoma was not observed in the transplantation of mESCs-derived DE cells by different treatment into mice with CCl_4-induced cirrhosis.

Table S1 Characterization of MSNs and modified MSNs

Sample name	DLS size(nm)	Zeta potential (mV)
MSNs	116.4±7.2	-45.1±6.2
PEI-MSNs	212.8±7.7	26.2±6.2
Activin A-PEI-MSNs	288.1±2.0	21.2±4.3
aFGF-PEI-MSNs	230.7±13.4	17.0±4.5
HGF-PEI-MSNs	267.7±3.0	24.1±8.7

The dynamic light scattering (DLS) sizes and zeta potential of MSNs, PEI-MSNs, Activin A-PEI-MSNs , aFGF-PEI-MSNs , HGF-PEI-MSNs in PBS buffer at pH 7.4 were measured by a ZetaSizer Nano (Malvern).

Table S2 Specific surface area (a_s,BET), total pore volume, and average pore diameters for MSNs and PEI-MSNs

**Sample**	**$a_{s, BET}$(m^2/g)**	**Vp(cm^3/g)**	**Dp(nm)**
MSNs	522.3	0.7	2.4
PEI-MSNs	329.8	0.7	2.1

Table S3 The sequences for primers of Q-PCR

**GenBank Accession No.**	**Gene name**	**Primer sequence**	**Product length (bp)**	**Annealing temperature (℃)**
NM_001291065.1	FoxA2	5'-ATCCGCCACTCTCTCTCCTT-3'<br>5'-TCTTCTTGCCTCCGCTACTG-3'	211	58
NM_001289464.1	Sox17	5'- CTCGGGGATGTAAAGGTGAA-3'<br>5'- CTTAGCTCTGCGTTGTGCAG-3'	182	57
NM_007423.4	Afp	5'- CCCAACCTTCCTGTCTCAGT-3'<br>5'- TCTCCTCGATGTGTTTCTGC-3'	136	57
NM_009654.3	Alb	5'- CAAGAGCCCGAAAGAAACGAA-3'<br>5'- AATAAGGATGTCTTCTGGCAAC-3'	166	57
NM_007824.2	Cyp7a1	5'- GTATGCCTTCTGCTACCGAGTG-3'<br>5'- CATGCGTAGACGGATCAGTTCA-3'	271	57
NM_001252569.1	Aat	5'- GGAATCACAGAGGAAAATGCTC-3'<br>5'- GATAGGGGGCATAGACATAGGAA-3'	135	58
NM_008061.3	G6p	5'- GGTATTTAAAGTCAACCGCCAT-3'<br>5'- CTCAGTTTCCAGCATTCACAC-3'	185	57
NM_001289726.1	GAPDH	5'-CATCAATGGAAATCCCATCA-3'<br>5'-TTCTCCATGGTGGTGAAGAC-3'	117	57

References

[1] D. M. Huang, T. H. Chung, Y. Hung, F. Lu, S. H. Wu, C. Y. Mou, M. Yao, Y. C. Chen, *Toxicol. Appl. Pharmacol.* **2008**, 231, 208.

[2] J. L. Vivero-Escoto, Slowing, II, B. G. Trewyn, V. S. Y. Lin, *Small* **2010**, 6, 1952.

[3] W. Chen, P. H. Tsai, Y. Hung, S. H. Chiou, C. Y. Mou, *ACS Nano* **2013**, 7, 8423.

[4] T. A. Xia, M. Kovochich, M. Liong, H. Meng, S. Kabehie, S. George, J. I. Zink, A. E. Nel, *ACS Nano* **2009**, 3, 3273.

[5] X. Li, Q. R. Xie, J. X. Zhang, W. L. Xia, H. C. Gu, *Biomaterials* **2011**, 32, 9546.

[6] S. Agarwal, K. L. Holton, R. Lanza, *Stem Cells* **2008**, 26, 1117.

[7] T. Teratani, H. Yamamoto, K. Aoyagi, H. Sasaki, A. Asari, G. Quinn, H. Sasaki, M. Terada, T. Ochiya, *Hepatology* **2005**, 41, 836.

[8] Y. Y. Duan, X. C. Ma, W. Zou, C. Wang, I. S. Bahbahan, T. P. Ahuja, V. Tolstikov, M. A. Zern, *Stem Cells* **2010**, 28, 674.

[9] X. L. Kuai, X. Q. Cong, X. L. Li, S. D. Xiao, *Liver Transplant.* **2003**, 9, 1094.

[10] F. M. Li, P. Y. Liu, C. C. Liu, D. Xiang, L. Deng, W. L. Li, K. Wangensteen, J. G. Song, Y. Ma, L. J. Hui, L. X. Wei, L. S. Li, X. Y. Ding, Y. P. Hu, Z. Y. He, X. Wang, *Gastroenterology* **2010**, 139, 2158.
